# Rotavirus Infection and Vaccination: Knowledge, Beliefs, and Behaviors among Parents in Italy

**DOI:** 10.3390/ijerph16101807

**Published:** 2019-05-21

**Authors:** Francesco Napolitano, Abdoulkader Ali Adou, Alessandra Vastola, Italo Francesco Angelillo

**Affiliations:** Department of Experimental Medicine, University of Campania “Luigi Vanvitelli”, Via L. Armanni, 5 80138 Naples, Italy; francesco.napolitano2@unicampania.it (F.N.); kadermed88@hotmail.it (A.A.A.); alexia291@hotmail.it (A.V.)

**Keywords:** behaviors, knowledge, parents, rotavirus vaccination, survey

## Abstract

This study was designed to investigate the knowledge, beliefs, and behaviors about rotavirus infection and its vaccination in a sample of parents in Naples, Italy. The survey was conducted between June and December 2018 among parents of children aged 3 months to 3 years. A total of 40.7% of the study subjects declared that they had heard about rotavirus infection and 60.8% and 59.2% were aware about the vaccination and of its availability in Italy. Parents with a child aged <1 year and those who reported the physicians as source of information were more likely to have heard about rotavirus infection and to know that the vaccination is available in Italy. More than half (56.4%) were worried that their children could have a rotavirus gastroenteritis and this was most likely to occur in those who have heard about rotavirus infection. Only 15.3% declared that they had immunized their children against rotavirus infection. Parents who considered it dangerous for their children to contract the rotavirus gastroenteritis, those who considered the rotavirus vaccine useful, and those who had received information by physicians were more likely to have vaccinated their children against the infection. More than half of the parents who did not immunize their children expressed their willingness to vaccinate them. Developing and implementing additional public education programs are needed for better knowledge toward rotavirus infection and vaccination and a high coverage among parents.

## 1. Introduction

Rotavirus is one of the leading causes of gastroenteritis among children younger than 5 years of age and data worldwide estimated that it globally caused approximately 37% of all diarrhea-associated hospitalizations and 215,000 deaths [1], whereas in Italy the estimated incidence is 5 cases per 100 [2].

The World Health Organization has recommended the introduction of the vaccine against rotavirus in all national immunization programs since 2009 [3,4]. In Italy, childhood vaccinations are provided by public centers and in Campania region rotavirus vaccination is recommended and free of charge since 2018 [5,6]. The schedule included two doses administered at 6 and 10 weeks of age with hexavalent vaccine (diphtheria-tetanus-pertussis, polio, hepatitis B, *H. influenzae*) and pneumococcal conjugate vaccine [5]. However, despite the vaccination’s implementation having resulted as a safe and effective public health strategy with a sustained reduction in rotavirus disease burden and deaths [7,8], coverage rates remain low in many countries [9]. In Italy, the vaccination uptake in 2018 was 16.5% for the 2015 birth cohort [10]. Primary care for children ≤6 years old is provided free of charge by primary care pediatricians and for those aged >6 years either by pediatricians or general practitioners.

The low coverage rates are still public health challenges and various factors have been cited for why parents and informal caregivers’ declined childhood vaccinations, such as vaccine hesitancy [11,12], lack of appropriate information [13,14], and perception of vaccine safety and benefits [15,16,17]. Therefore, an understanding of the parents’ knowledge, beliefs, and behaviors regarding vaccination against rotavirus could provide useful information on the factors that influence the uptake and to design and to develop interventions to increase the coverage. To date, there have been few attempts worldwide on what parents know, beliefs, and experiences on this topic [18,19,20], while there is a knowledge gap in this geographic area of Italy with the attention on different groups mainly focused on Human Papillomavirus (HPV) [21,22,23,24,25] and on vulnerable populations [17,26,27]. Therefore, the present cross-sectional study was designed to investigate the knowledge, beliefs, and behaviors about rotavirus infection and the relative childhood vaccination and to investigate their determinants in a sample of parents in Italy.

## 2. Materials and Methods

### 2.1. Settings and Participants

Data collection occurred between June and December 2018 in the geographic area of Naples, Italy. The population of interest was parents of children aged from 3 months to 3 years of age. A one-stage cluster sample has been used. From the list of all public nurseries, eight of them were selected using a computer-generated list of random numbers, and all children attendees were recruited. In Italy, children can attend nurseries from three months of age. The sample size calculation was based on the expected positive attitude of parents towards the willingness to vaccinate their children with rotavirus vaccine of 80% [19,20], a confidence interval of 95%, and an error of 5%. Assuming a response rate of 80%, the final sample size was calculated to be of 307 individuals.

### 2.2. Procedure

Before starting the study, the research team contacted by letter the heads of the selected nurseries and asked the approval to conduct the survey. The heads received information about the study’s purposes and procedure. After getting the permission, two researchers contacted the teachers of each class, who delivered to all parents a sealed envelope that contained a letter with information regarding the purposes of the study, an informed consent form, a questionnaire, and an envelope to return the completed questionnaire along with the signed consent form. In the letter it was also made clear that completing the questionnaire was voluntary and that all of information collected would be processed anonymously and confidentially. Those who agreed to participate completed the questionnaire at home. Only one parent of each child filled out the questionnaire. After three weeks, the researchers contacted the teachers to collect the questionnaires. No monetary compensation or gift has been given to the surveyed parents.

### 2.3. Questionnaire

The structured self-administered questionnaire developed by the Authors comprised 26 items grouped into the following five sections: (a) socio-demographic characteristics (gender, age, nationality, marital status, education, employment status, husband/wife/partner education and employment status, number and ages of children); (b) knowledge regarding the rotavirus infection and the relative vaccination (having heard about the infection, clinical symptoms, modes of transmission, preventive measures, availability of the vaccination against rotavirus in Italy); (c) attitudes about rotavirus gastroenteritis and related vaccination (preoccupation that their children may acquire the infection, concern about the dangerousness of the infection, agree that the vaccine is important to protect their children, concern about the dangerousness of the vaccination, willingness or unwillingness to vaccinate their children); (d) behaviors regarding vaccination (whether or not they had vaccinated their children); and (e) trusted rotavirus infection and vaccination information sources. The questions regarding the knowledge had the response yes, no, or do not know. A ten-point Likert scale was used for questions regarding beliefs, with possible responses ranging from “1” to “10” with higher values corresponding to a stronger attitude. The remaining questions had multiple-choice alternatives.

The developed questionnaire was assessed in a pilot involving 20 mothers of children of vaccination age prior to administering it to the study population, in order to ensure that the questions were understood as intended, and to omit questions that were misinterpreted.

The study was approved by the Ethics Committee of the Teaching Hospital of the University of Campania “Luigi Vanvitelli” (approval number 484).

### 2.4. Statistical Analysis

Data were analyzed using STATA version 15, StataCorp LLC: College Station, TX, USA [28]. The first level of analysis comprised a descriptive analysis of the responses. The second level of analysis involved univariate tests of association. Chi-square for categorical variables and Student’s *t*-test for continuous variables were used to check whether there was an association between the outcomes of interest and the independent variables. Those determinants that showed an association with a *p*-value less or equal than 0.25 with the outcomes of interest in the univariate analyses were entered simultaneously in the multivariate model to assess their unique contribution to the explanation of the outcomes. The third level of analysis included multivariate stepwise logistic regression performed to identify the association of independent characteristics with the following outcomes of interest: Having heard about the rotavirus infection and having the knowledge that the vaccination against rotavirus is available in Italy (Model 1), parents who were very worried that their children could have a rotavirus gastroenteritis (Model 2), and having immunized their children against the rotavirus infection (Model 3). The following variables were included in all Models: Age (<40 years = 0; ≥40 years = 1), gender (male = 0; female = 1), educational level (none or primary/middle/high schools = 0; college degree or higher = 1), at least one parent who is a healthcare worker (no = 0; yes = 1), having a child aged <1 year (no = 0; yes = 1), having more than one child (no = 0; yes = 1), having received information on rotavirus infection and relative vaccination from physicians (no = 0; yes = 1), need to receive additional information about rotavirus infection and relative vaccination (no = 0; yes = 1). Moreover, the following variables were included in Models 2 and 3: Having heard about rotavirus infection (no = 0; yes = 1) and having the knowledge that the vaccination against rotavirus is available in Italy (no = 0; yes = 1). Finally, the following variables were included in Model 3: Being very worried that their children could have a rotavirus gastroenteritis (no = 0; yes = 1), considering dangerous for their children to contract the rotavirus gastroenteritis (continuous), considering useful the rotavirus vaccine (continuous), and considering dangerous for their children the rotavirus vaccine (continuous).

The significance level for variables entering in the stepwise logistic regression models was set at 0.2 and for removing at 0.4. Odds ratios (ORs) were calculated along with their associated 95% confidence intervals (CIs) in the multivariate logistic regression analysis. All tests were two-tailed and the results were evaluated at a significance level of 0.05 or less.

## 3. Results

### 3.1. Participants

In total, 500 individuals were approached to participate and 307 consented and were enrolled in the survey for a response rate of 61.4%. Selected characteristics of the parents who participated in the study are presented in Table 1. Only 4.3% had ≤25 years, the majority (52.9%) were aged between 26 and 34, 42.8% had ≥35 years with an overall mean and median age of 33.5 and 34, respectively. The respondents were predominately female, more than two thirds were married, 55.9% have more than one child, one third had a college degree or above, and two thirds were employed.

### 3.2. Knowledge

The results regarding the knowledge about rotavirus infection of the study population are presented in Table 2. Less than half declared that they had heard about rotavirus infection (40.7%), and similar values had been observed among those who had a child aged <1 year (40.9%) and with more than one child (40.3%). More than half of those who had heard about rotavirus infection were aware that it can be transmitted by contaminated water (60.8%) and foods (56%), and more than one third by contact with contaminated surfaces (38.4%) and through person-to-person (36%). Regarding the vaccination, 60.8% and 59.2% were aware about it and of the availability in Italy, 44.1% of them knew that there are two vaccines (two or three doses schedule) for children, but none indicated correctly the schedule. Univariate analysis showed that participants with a college degree or above (*p* = 0.001) and those who have received information about rotavirus infection and vaccination by physicians (*p* < 0.001) have heard about rotavirus infection and know that the vaccination is available in Italy. Table 3 showed the multivariate stepwise logistic regression analysis results on the potential factors affecting the different outcomes of interest. The results demonstrated that only two determinants were significantly associated with the knowledge. Indeed, parents with a child aged <1 year and those who reported the physicians as source of information were almost 3 (95% CI 1.42–7.36) and 21 times (95% CI 9.84–46.17) respectively more likely to have heard about rotavirus infection and to know that the vaccination is available in Italy (Model 1).

### 3.3. Beliefs

The results regarding parents’ beliefs about rotavirus infection are presented in Table 4. More than half (56.4%) were worried that their children could have a rotavirus gastroenteritis and two thirds (63.7%) considered dangerous for their children to contract it with a mean value respectively of 6 and 6.4, out of a maximum score of 10. Almost two thirds (60.8%) considered that the rotavirus vaccine was important for their child with an average value of 6.5 and the vast majority (89%) believed that it was not harmful. In the bivariate analysis, having heard about rotavirus infection (*p* < 0.001) and having received information from physicians (*p* < 0.001) were significantly associated with the parents’ worrying that their children could have a rotavirus gastroenteritis. The multivariate stepwise logistic regression analysis confirmed that parents who have heard about rotavirus infection (OR = 6.47; 95% CI 2.56–16.41) were more likely to be very worried that their children could have a rotavirus gastroenteritis compared with those who did not have this knowledge (Model 2 in Table 3).

### 3.4. Behaviors

The results regarding the parents’ behaviors about rotavirus infection are reported in Table 5. Only 15.3% of the total sample and 15% of those who had more than one child declared that they had immunized their children against rotavirus infection. The bivariate analysis revealed that ten variables were significantly associated with this behavior: Lower age (*p* = 0.015), having a college degree or above (*p* < 0.001), having heard about rotavirus infection (*p* < 0.001), having heard about rotavirus vaccination (*p* < 0.001), worry that their children could have a rotavirus gastroenteritis (*p* < 0.001), considering dangerous for their children to contract a rotavirus gastroenteritis (*p* < 0.001), considering useful the rotavirus vaccine (*p* < 0.001), believing that the vaccine was not harmful for their children (*p* < 0.001), having received information about rotavirus infection and relative vaccination by physicians (*p* < 0.001), and needing more information (*p* < 0.001). After adjustment for the potential confounding variables, logistic regression analysis showed that the variables that were significantly associated with a higher likelihood of having their children vaccinated against rotavirus infection were considering it dangerous for their children to contract a rotavirus gastroenteritis (OR = 1.91; 95% CI 1.25–2.91), having positive perception toward the effectiveness of the vaccine (OR = 1.45; 95% CI 1.02–2.07), and having received information about rotavirus infection and relative vaccination by physicians (OR = 25.09; 95% CI 6.52–96.61) (Model 3 in Table 3). Reasons for having immunized their children were: Wanted to reduce the chance of getting the disease (76.7%), had been told to do so by the pediatricians (75%), and fear that the infection could cause severe health problems (63.8%). The most frequent reasons for those who did not immunize their children were: Lack of knowledge about vaccination (77.9%), the vaccination had not been recommended by the pediatrician (31.6%), and concerns about the side effects of the vaccine (15.8%). Among those who did not immunize their children, more than half of the total sample (51.3%) and of those who had more than one child (52.7%) expressed their willingness to vaccinate their children. The most frequently selected reasons for this positive attitude were that the rotavirus vaccination is useful (55.7%), the infection could cause severe health problems (44.7%), and a recommendation by the pediatrician (20.5%). Reasons for being unwilling to immunize included lack of knowledge about vaccination (53.6%), concerns about the side effects of the vaccine (30.4%), and lack of recommendation by the pediatrician (30.1%).

### 3.5. Information Sources

The most common sources of rotavirus infection and vaccination-related information were physicians (68%) and internet (63.9%), followed by media/advertisement (25.5%), and family (15.5%). Finally, two-thirds of the respondents (63.5%) wished to receive more information about vaccines.

## 4. Discussion

This study, conducted within the season when rotaviruses do not peak in temperate countries, is the first to specifically analyze the knowledge, beliefs, and behaviors towards rotavirus infection and relative vaccination for their children and of their influencing predictors in the population of parents in Italy. The results have several implications considering the Italian Immunization Prevention Plan 2017–2019, the recent legislation on mandatory and recommended vaccinations, and the low coverage for rotavirus vaccination in children that has been reported in Italy.

First, a striking observation in the present results was the low levels of knowledge regarding rotavirus infection, how it can be transmitted, and also about the vaccination currently available. Indeed, less than half had heard of the infection and only 59.2% of them knew that the vaccine was available in Italy. This lack of knowledge indicates that comprehensive information about rotavirus infection and relative vaccination is not being widely disseminated and this is a very worrying result because it is well known that rotavirus gastroenteritis could have serious health complications for children. Therefore, it is urgent to improve the level of knowledge of the population through effective pediatricians recommendation of vaccine and educational interventions using all occasions in which it is possible to meet the population interested in vaccination such as, for example, prenatal-classes or parents’ utilization of the vaccinations service for their children. Moreover, parents could benefit from targeted communication strategies and promotion of vaccination education programs because effective physician-patient communication may increase the vaccines’ knowledge, clarifies the concerns, and motivates the population to vaccinations acceptability [29,30,31].

Second, more than half were worried that their children could have a rotavirus gastroenteritis, two thirds considered it dangerous for their children to contract it, and the majority had positive attitudes towards rotavirus vaccination since almost two thirds considered that it is important for their child. Although the majority of participants (89%) agreed with the statement that the vaccine was not harmful, among those who did not immunize their children a large proportion reported that they will not be willing to obtain vaccination mainly because a lack of knowledge or concerns of side effects. This underlines the importance of health education towards targeting population, especially those with common misconceptions regarding vaccinations, to eliminate their worries about safety. Moreover, an association has been observed between knowledge and attitudes, since respondents who very worried that their children could have rotavirus gastroenteritis were those who have heard about rotavirus infection. This finding is in keeping with findings of previous studies that showed that vaccination might be accepted in those more knowledgeable [26,32,33]. These findings indicate that providing specific vaccine-related knowledge may help to change their attitudes.

Third, a concerning result from this study was, not surprisingly, the insufficient vaccination rate with only 15.3% of the participants reported having immunized their children against rotavirus infection. This result, in line with the rate in Italy [10], highlights the need for greater efforts by policymakers and pediatricians to recommend vaccination more effectively in order to improve the coverage. Moreover, the results showed that parents had high intention to get their children vaccinated in spite of the low uptake rate. As expected, those considering it dangerous for their children to contract rotavirus gastroenteritis and having positive perception toward the effectiveness of the vaccine were more likely to have immunized their children. These findings are consistent with several studies worldwide evaluating factors positively associated with vaccination [15,17,20,33,34,35]. These observations underlined the importance of not only adequately providing information about rotavirus infection to parents, but also explaining the utility of the vaccination in order to increase the coverage.

Fourth, the results of the multivariable regression analysis indicated other several interesting associations. The exposure to sources of information had a significant effect on rotavirus infection and vaccination-related knowledge and uptake. However, not all sources were equally effective. Pediatrician was the strongest predictor for the reported knowledge and child vaccination status: Parents who reported this source of information were 21 times more likely to have heard about rotavirus infection and to know that the vaccination is available in Italy and 25 times to have a child vaccinated against rotavirus. These findings corroborate previous research on the key role that they play in promoting vaccinations. Notably, having received information from healthcare professionals had a significantly stronger association with the level of knowledge and the vaccination rates [17,22,27,36,37,38]. This is a clear indication of the need to develop and to disseminate interventions to improve the interaction between pediatrician and all eligible patients, although more than two-thirds of participants identified pediatricians as a primary source of information. Similar results were also seen in previous studies [39,40,41,42]. Moreover, the majority of the participants indicated an interest in receiving additional information regarding vaccines, implying that this gap may not necessarily due to parent disinterest. The interventions should also address parents’ barriers. For example, insufficient knowledge about vaccination, concerns regarding the side effects, and the lack of recommendation by the pediatrician were the most frequently cited barriers for not having immunized their children. The lack of recommendation by pediatricians underline the need that they should be aware of their role in providing adequate, clear, and accessible information and communications to parents regarding rotavirus infection, vaccine safety, and adverse effects, since this may be one way to reduce concerns and misconceptions. Moreover, almost two-thirds of the sample cited Internet as source of information and this may be of concern due the spread of anti-vaccine arguments online and this may be translated into lower vaccine coverage. Monitoring social media for anti-vaccine beliefs is essential and effective health communication must combat the anti-vaccination campaigns. Websites that address public health issues should provide clear and evidence-based information easily accessible to users.

This study has some methodological limitations that warrant discussion and the results must be interpreted and used with caution. Firstly, the survey was conducted in one geographical area and thus, the generalizability of the findings to the rest of Italy needs to be established. However, we are confident that the characteristics of the sample are similar to those of other areas of the country and the observed vaccination coverage is in line with the value throughout the whole country [10]. Secondly, the recruitment has been performed through public nurseries, and, therefore, parents of children who do not attend any pre-schools or private nurseries were excluded. It is well known that those attending public schools may belong to lower social groups and, therefore, they may not be representative of the parents’ population. However, we believe that this would not have an impact on the conclusions of the study, although we cannot be certain, because attending pediatricians and preventive healthcare services is provided free of charge. Thirdly, caution should be taken when interpreting the findings owing to the cross-sectional study that allowed for identification of associations and prevent from making any statements regarding causality. Fourthly, the survey data, including child vaccination status, were collected based on self-reporting and were not verified by a certification and, therefore, the information is susceptible to forgetting and recall bias that may have resulted in an underestimation of vaccination. Moreover, the possible association of rotavirus vaccine with another multivalent vaccine may lead to some parents not realizing that their children have received it. The inclusion of parents of very young children may have reduced the bias. Fifthly, the possibility of the parents responding in a socially expected way and this would mean that the reported intentions would not correspond to the real situation or their future behaviors. However, the fact that the questionnaires were self-administered and completed anonymously should result in reducing this bias.

## 5. Conclusions

The current survey indicated low levels of knowledge and coverage on rotavirus vaccination and highlight the need for developing and implementing additional public education programs so that parents are informed, which should result in a better knowledge toward rotavirus infection and vaccination and in a high coverage.

## Figures and Tables

**Table 1:** Main characteristics of the study respondents.

	Total (*n* = 307)	
	*n*	%
Gender		
Female	257	84.5
Male	47	15.5
Age (years)	33.5 ± 4.9 (18–47) *	
Marital status		
Married	250	82.3
Other	54	17.7
Educational level (years)		
None/Primary school (<9)	4	1.4
Middle school (9–11)	54	17.9
High school (12–16)	158	52.5
College degree or higher (>16)	85	28.2
Employment status		
Employed	211	69.9
Unemployed	91	30.1
At least one parent who is a healthcare professional		
No	280	96.2
Yes	11	3.8
Number of children		
1	135	44.1
≥2	171	55.9

Number for each item may not add up to total number of study population due to missing value; * Mean ± Standard deviation (Range).

**Table 2:** Knowledge about rotavirus infection of the study respondents.

	***n***	**%**
Have heard about the rotavirus infection ^a^	125 ÷ 307	40.7
Knowledge that the vaccination against rotavirus is a preventive measure ^b^	76 ÷ 125	60.8
Knowledge that the rotavirus vaccine is available in Italy ^b^	74 ÷ 125	59.2
	Correct response	
Modes of transmission of rotavirus ^b^	*n*	%
Contaminated water (true)	76 ÷ 125	60.8
Contaminated food (true)	70 ÷ 125	56
Contaminated surfaces (true)	48 ÷ 125	38.4
Person-to-person (true)	45 ÷ 125	36

^a^ All sample (*n* = 307); ^b^ Only for those who reported that they have heard about rotavirus infection.

**Table 3:** Multivariate analysis results.

Variable	OR	SE	95% CI	*p*
**Model 1. Having heard about rotavirus infection and having the knowledge that the vaccination against rotavirus is available in Italy**				
Log-likelihood = −109.22, χ^2^ = 86.37, *p* value < 0.0001				
Parents who received information on rotavirus infection and relative vaccination from physicians	21.31	8.4	9.84–46.17	<0.001
Having a child aged <1 year	3.23	1.35	1.42–7.36	0.005
Having more than one child	0.55	0.2	0.26–1.13	0.106
Age ≥40 years	2.2	1.12	0.82–5.95	0.119
At least one parent who is a healthcare worker	3.49	2.94	0.67–18.19	0.137
Female	Backward elimination			
Having college degree or higher level of education	Backward elimination			
Need to receive additional information	Backward elimination			
**Model 2. Parents who were very worried that their children could have a rotavirus gastroenteritis**				
Log-likelihood = −91.44, χ^2^ = 19.2, *p* value = 0.003				
Having heard about rotavirus infection	6.47	3.07	2.56–16.41	<0.001
Having more than one child	0.57	0.23	0.26–1.27	0.172
Having the knowledge that the vaccination against rotavirus is available in Italy	0.51	0.27	0.18–1.45	0.211
Parents who received information on rotavirus infection and relative vaccination from physicians	0.57	0.32	0.19–1.71	0.320
Having a child aged <1 year	1.58	0.73	0.64–3.93	0.322
Age <40 years	0.49	0.39	0.11–2.29	0.369
Female	Backward elimination			
Need to receive additional information	Backward elimination			
**Model 3. Having immunized their children against the rotavirus infection**				
Log-likelihood = −45.82, χ^2^ = 150.18, *p* value < 0.0001				
Parents who received information on rotavirus infection and relative vaccination from physicians	25.09	17.25	6.52–96.61	<0.001
Parents who considered dangerous for their children to contract the rotavirus gastroenteritis	1.91	0.41	1.25–2.91	0.003
Parents who considered useful the rotavirus vaccine	1.45	0.26	1.02–2.07	0.037
Parents who were very worried that their children could have a rotavirus gastroenteritis	0.81	0.12	0.65–1.12	0.15
Age ≥40 years	4.56	4.45	0.67–30.83	0.119
Having heard about rotavirus vaccine	2.34	1.68	0.57–9.66	0.239
Parents who considered dangerous for their children the rotavirus vaccine	0.86	0.12	0.65–1.13	0.271
Having a child aged <1 year	2.34	1.87	0.49–11.21	0.286
Having more than one child	0.54	0.33	0.16–1.81	0.319
Need to receive additional information	0.57	0.33	0.18–1.81	0.341
Having heard about rotavirus infection	2.41	2.39	0.34–16.95	0.378
Having college degree or higher level of education	Backward elimination			

**Table 4:** Beliefs about rotavirus vaccination of the study respondents.

	*n*	%
Parents who believed that the vaccine was not harmful for their children	259 ÷ 291	89
Parents who considered dangerous for their children to contract the rotavirus gastroenteritis	191 ÷ 300	63.7
Parents who considered useful the rotavirus vaccine	178 ÷ 293	60.8
Parents who were worried that their children could have a rotavirus gastroenteritis	172 ÷ 305	56.4

Number for each item may not add up to total number of study population due to missing value.

**Table 5:** Behaviors about rotavirus vaccination of the study respondents.

	*n*	%
Having immunized their children against the rotavirus infection	47 ÷ 307	15.3
Reasons for having immunized their children		
Wanted to reduce the chance of getting the disease	36 ÷ 47	76.7
Had been told to do so by the pediatricians	35 ÷ 47	75
Fear that the infection could cause severe health problems	30 ÷ 47	63.8
Reasons for not having immunized their children		
Lack of knowledge about the vaccination	202 ÷ 259	77.9
Vaccination had not been recommended by the pediatrician	93 ÷ 258	36.1
Concerns about the side effects of the vaccine	41 ÷ 260	15.8

Number for each item may not add up to total number of study population due to missing value.
